# Test-retest, intra- and inter-rater reliability of the reactive balance test in patients with chronic ankle instability

**DOI:** 10.3389/fneur.2024.1320043

**Published:** 2024-02-16

**Authors:** Alexandre Maricot, Elke Lathouwers, Jo Verschueren, Kevin De Pauw, Romain Meeusen, Bart Roelands, Bruno Tassignon

**Affiliations:** ^1^Human Physiology and Sports Physiotherapy Research Group, Faculty of Physical Education and Physiotherapy, Vrije Universiteit Brussel, Brussels, Belgium; ^2^Brussels Human Robotics Research Center (BruBotics), Vrije Universiteit Brussel, Brussels, Belgium; ^3^Department of Sports, Recreation, Exercise and Sciences (SRES), Faculty of Community and Health Sciences, University of the Western Cape, Cape Town, South Africa

**Keywords:** ankle injury, screening, neurocognitive performance test, reproducibility, functional performance test

## Abstract

**Introduction:**

The Reactive Balance Test (RBT) could be a valuable addition to research on chronic ankle instability (CAI) and clinical practice, but before it can be used in clinical practice it needs to be reliable. It has already been proven reliable in healthy recreational athletes, but not yet in patients with CAI who have shown persistent deficits in dynamic balance. The study aimed to determine the test-retest, intra-, and inter-rater reliability of the RBT in patients with CAI, and the test-retest and inter-rater reliability of the newly developed RBT score sheet.

**Methods:**

We used a repeated-measures, single-group design to administer the RBT to CAI patients on three occasions, scored by multiple raters. We included 27 participants with CAI. The study used multiple reliability measures, including Pearson r, intra-class correlations (ICC), standard error of measurement (SEM), standard error of prediction (SEP), minimal detectable change (MDC), and Bland–Altman plots, to evaluate the reliability of the RBT’s outcome measures (visuomotor response time and accuracy). It also assessed the test-retest and inter-rater reliability of the RBT score sheet using the same measures.

**Results:**

The ICC measures for test-retest reliability were similar for accuracy (0.609) and VMRT (0.594). Intra-rater reliability had high correlations and ICCs for accuracy (*r* = 0.816, ICC = 0.815) and VMRT (*r* = 0.802, ICC = 0.800). Inter-rater reliability had a higher ICC for VMRT (0.868) than for accuracy (0.690).

**Conclusion:**

Test-retest reliability was moderate, intra-rater reliability was good, and inter-rater reliability showed moderate reliability for accuracy and good reliability for VMRT. Additionally, the RBT shows robust SEM and mean difference measures. The score sheet method also demonstrated moderate test-retest reliability, while inter-rater reliability was good to excellent. This suggests that the RBT can be a valuable tool in assessing and monitoring balance in patients with CAI.

## 1 Introduction

Chronic ankle instability (CAI) is a multifaceted clinical condition affecting approximately 40–45% of the adult population who have experienced a primary ankle sprain (1–3). The main symptoms of CAI are the prolonged symptoms of self-reported disability, local neuromuscular deficits and recurrent episodes of the ankle “giving way” (4–11). CAI negatively impacts health-related quality of life (12–16) and may alter physical activity levels (17–21). Moreover, an association between CAI and an early onset of osteoarthritis has been established (22–27). Due to the prevalence and long-lasting impairments of CAI, it is essential to develop testing tools to identify those at risk of (re) injury, to monitor rehabilitation progress and to make better-informed return to sport (RTS) decisions (28–34).

For these purposes, clinicians often use functional performance tests (FPTs) (28–31, 35). Two of the most reliable FPTs with excellent criterion validity for assessing dynamic balance of the lower extremities are the star excursion balance test (SEBT) and the Y-balance test (YBT) (35–40). Poor performance on these tests is associated with an increase in lower extremity injury risk (35, 37, 40–43). The SEBT measures maximum reach distance in eight directions whilst maintaining single-leg balance (44). A shorter and more reliable version, the YBT (45) uses the sum of three reach directions (anterior, posteromedial and posterolateral) to assess injury risk and identify patients with CAI (35, 41–43). Typically, individuals with CAI demonstrate a significantly lower reach distance on both FPTs (46–57).

However, The SEBT and YBT are limited in their ability to be applied to on-field sports contexts because they involve pre-planned tasks without considering dynamic environmental aspects of sports practice (35, 58). Functional exercises with neurocognitive components like decision-making and reaction times, in tandem with dynamic balance requiring an external focus of attention, are essential factors in sports injury risk and performance in sports such as basketball, football and table tennis (58–65).

Numerous studies have discovered that individuals with CAI have worse dynamic balance when performing tasks simultaneously than healthy individuals, suggesting that they require more attentional resources to stay balanced (66–75). Neurocognitive deficits, particularly reduced spatial awareness, have been observed in people with CAI assessed through computerized neurocognitive tests (76–78). This decline in spatial awareness may negatively impact their capacity to respond to environmental obstacles. Patients with CAI may consequently display less accuracy or slower reaction times when performing neurocognitive tasks that call for quick thinking and spatial awareness explaining these deficits.

The reactive balance test (RBT) was developed (79) to add neurocognitive components such as decision-making, visuomotor responses and environmental perception to the YBT in a standardized way. The RBT’s primary outcome measures involve visuomotor response time (VMRT) and accuracy (79). Moreover, the RBT could be a valuable addition to research on CAI and clinical practice, but before it can be used in clinical practice it needs to be reliable. It has already been proven to reliably measure accuracy and VMRT in healthy recreational athletes (80), but not yet in patients with CAI. Therefore, the primary aim of this study was to determine the test-retest intra- and inter-rater reliability of the RBT in these patients through video-based assessment. The secondary objective of this study was to determine the test-retest, and inter-rater reliability of the RBT using a score sheet to assess the patients’ accuracy in real-time. The score sheet was designed to enable rapid and efficient data collection, enabling evaluators to swiftly assess RBT performance without requiring time-consuming video playback. This approach could prove particularly beneficial when clinicians are working with extensive sample sizes or time-sensitive evaluations.

## 2 Materials and methods

We applied the guidelines for reporting reliability and agreement studies (GRRAS) (81) to report the RBT’s reliability in individuals with CAI. We wanted to evaluate the reliability of the video-based analyzes and newly designed score sheet we developed. The video-based analysis entails the reviewer going through each trial afterward and scoring the RBT performance manually. In an effort to enhance efficiency and cut down on expenses, we introduced a score sheet that allows real-time scoring of the RBT performance. The consistency of the RBT outcome measures over time, under repeated and similar conditions, was defined as the test-retest reliability. The inter-rater reliability refers to how consistently different raters determine the accuracy and VMRT of patients of the same experimental trial. The intra-rater reliability of the RBT shows how consistently a rater can determine both RBT outcomes (82). The protocol was approved by the ethics committee of the UZ Brussel/Vrije Universiteit Brussel (B.U.N. 1,432,021,000,658) and was carried out following the “Code of Ethics of the World Medical Association” (Declaration of Helsinki).

### 2.1 Participants

Sample size calculations were based on the mathematics of Walter et al. (83). A reliability study with two experimental trials (*n* = 2), a null hypothesis of 0.5 and an alternative hypothesis of 0.8 based on an alpha of 0.05 and a beta error probability of 0.20 would require at least 22 participants. This reliability study included 27 individuals with CAI. Participants were recruited using convenience sampling through the Vrije Universiteit Brussel internal network and the social network of the researchers. Before participating in this study, all participants had to sign the informed consent form and complete three questionnaires Cumberland Ankle Instability Tool (CAIT), Foot and Ankle Ability Measure (FAAM) and the International Physical Activity Questionnaire (IPAQ). Depending on their MET/week the IPAQ assigns the participant to one of three classes: low physical activity level, moderate physical activity level and high physical activity level (84). Participant characteristics can be found in Table 1.

**Table 1:** Patient characteristics.

Type	Mean ± SD	95% CI	Min	Max
Age (years)	22 (± 2.3)	21–23	18	28
Height (cm)	175.8 (± 10.3)	171.8–179.9	160	194
Weight (kg)	71.4 (± 10.1)	64.7–75.4	50.0	94.8
CAIT score	16.6 (± 3.6)	15.1–18.0	9	22
FAAM-ADL (%)	87.9 (± 11.3)	83.4–92.4	58	100
FAAM-S (%)	79.4 (± 15.4)	73.3–85.6	38	100
IPAq-score (MET/week)	4,560 (± 2,322)	3,641–5,478	1,080	9,172

ADL, activities of daily living; CAIT, Cumberland Ankle Instability Tool; FAAM, Foot and Ankle Ability Measure; IPAQ, International Physical Activity Questionnaire; MAX, maximum; MIN, minimum; S, Sport; SD, standard deviation; 95% CI, 95% Confidence Interval.

All participants were informed of the nature and procedures of the study. In- and exclusion criteria were based on the International Ankle Consortium guidelines (85). Forty-three eligible patients were screened on the following inclusion criteria: they (1) were between 18 and 35 years of age, (2) participate in at least one sport or physical workout at least once a week, (3) have a history of at least one significant ankle sprain that occurred at least 12 months before study enrolment that was associated with inflammatory symptoms (e.g., pain and swelling), (4) their initial sprain created at least one interrupted day of desired physical activity and (5) their most recent injury must have occurred more than 3 months before study enrolment, (6) experience recurrent sprains and ‘feelings of giving way’, (7) have at least two episodes of giving way in the 6 months before study enrolment, (8) and have a CAIT score of <24. All patients did not receive treatment during the study.

Potential participants with (1) a history of a fracture, (2) previous surgery to musculoskeletal structures (i.e., bones, joint structures, nerves) in the previous 2 years in either lower limb, (3) or any other relevant medical history, treatment or current condition (such as neurological diseases, inner ear disorders, color blindness etc.), which could affect balance ability or the action-perception pathways were excluded. Further exclusion criteria were evaluated in the pre-test checklist. All subjects were asked to limit alcohol and caffeine consumption the before and the day of each trial, not to take performance-enhancing medications, not to perform vigorous physical activities 24 h before the trial and to sleep at least 7 h the night before the trials. All participants confirmed to comply with the given instructions by completing the pre-test checklist.

### 2.2 Procedures

#### 2.2.1 Test protocol

**Figure 1:**
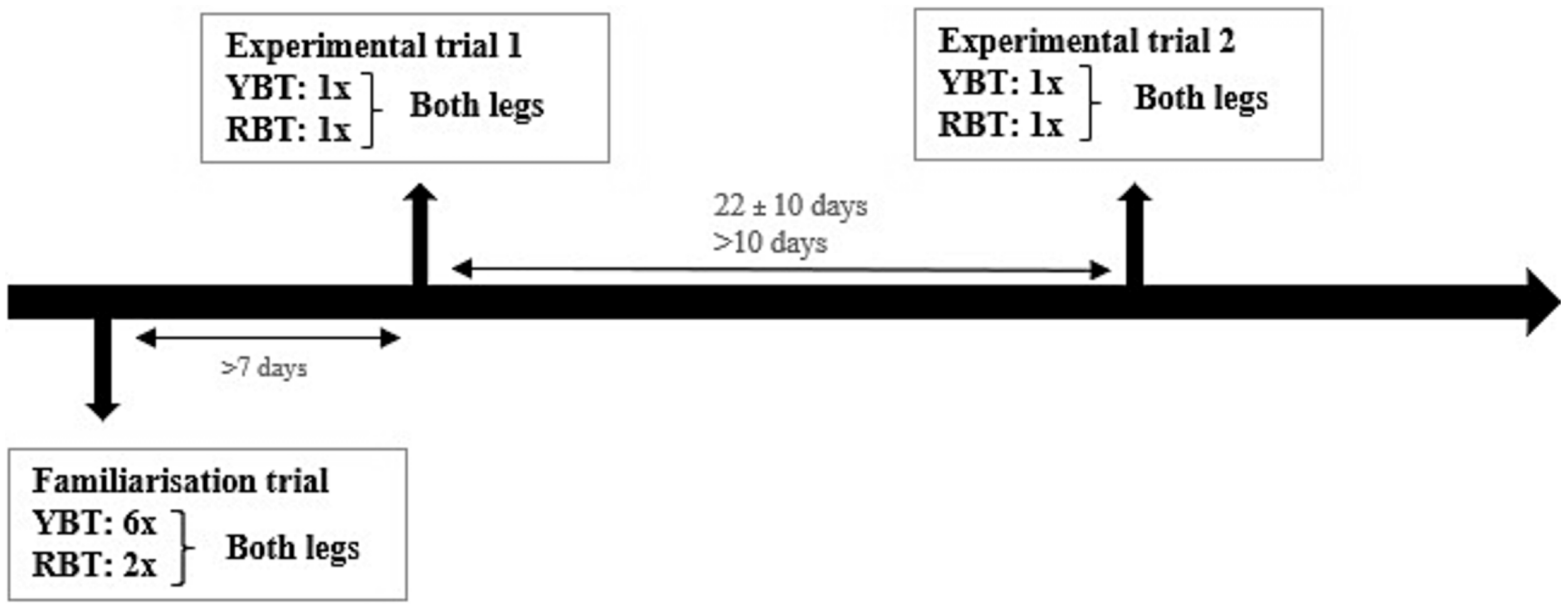
Test protocol; RBT, Reactive Balance Test; YBT, Y-Balance Test.

Patients visited the laboratory three times, once for a familiarization trial (± 1 h30) and twice for identical experimental trials (± 30 min). During the familiarization trial, the YBT was performed six times and the RBT two times on both legs. In each experimental trial, the YBT and the RBT were performed on both legs once. Figure 1 depicts an overview of a subject’s participation protocol and timeline. The familiarization was conducted at least 1 week prior to the first experimental trial to ensure that participants had adequate time to get acquainted with the test procedures and to attenuate possible learning effects during the experimental trials. The duration and range of the test-retest time frame were chosen to reflect a clinically relevant period and lasted 22 (± 10) days on average (86). During the measurements, it was ensured that the conditions were always the same.

#### 2.2.2 Y-balance test (YBT)

The Y-Balance Test (YBT Kit^™^, FunctionalMovement.com, Danville, VA) was performed on the Y-balance test kit. The YBT was performed according to the protocol and instructions described in earlier reliability research (40, 45, 80) provided guidelines for scoring and evaluating the test results. The test determines the subject’s maximum reach distance and ability to maintain balance while reaching in different directions. The maximal reach distance was measured by reading the reach distance at the proximal edge of the reach indicator. A reach was considered successful if the participant followed the instructions of Plisky et al. (45) and Gribble et al. (40). The trial was discarded and repeated if the subject; failed to maintain a one-sided stance on the platform, failed to maintain foot contact with the reach indicator on the target area while it was in motion (e.g., kicked the reach indicator), used the reach indicator for support of the stance, did not return to the starting position under control, or failed to keep hands on the iliac crest. The YBT was performed three times in each direction.

#### 2.2.3 Reactive balance test (RBT)

**Figure 2:**
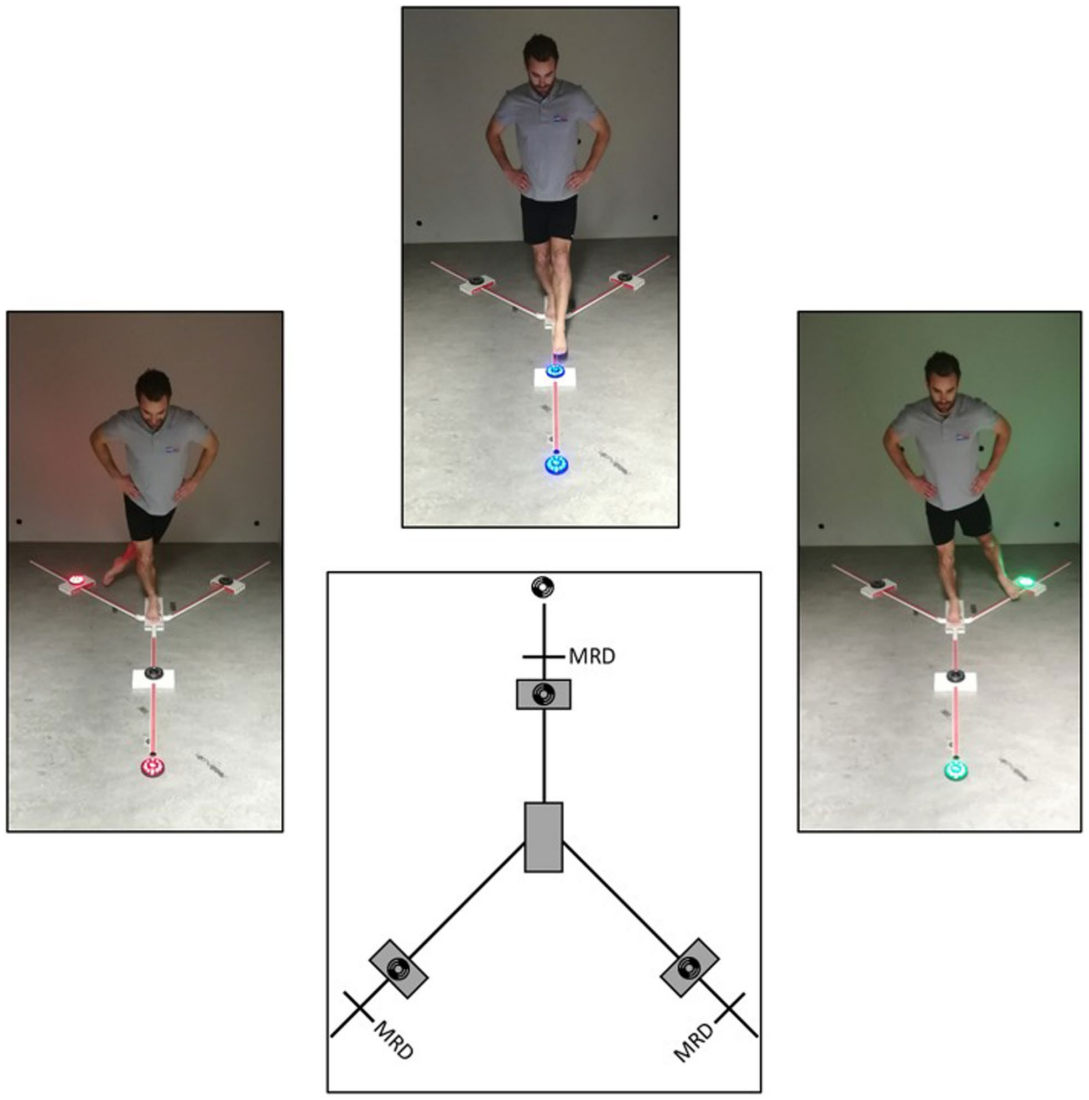
Reactive balance test. Reprinted with permission from Verschueren et al. (79); MRD, Maximal reach distance.

The RBT is neurocognitive FPT that assesses VMRT and dynamic balance using visual stimuli. Its purpose is to extinguish a color-matched LED light on the YBT in response to an initial leading visual stimulus (Figure 2). The protocol, procedures, and scoring system are explained in detail in the study of Verschueren et al. (79).

The test uses Fitlight^™^ hardware, software, and four LED lights placed on the YBT Kit^™^. LED lights are positioned on each corresponding axis of the Y-balance Test Kit™ using 80% of the maximum reach distance plus six centimeters, as six centimeter corresponds with the radius of the LED lights housing. Each color is assigned to a specific axis and appears 12 times at random for 36 stimuli, with inter-stimulus times varying between 1.5, 2, and 2.5 s. Blue represented the front axis, green the posteromedial, and red the posterolateral. The Fitlight^™^ software were programmed to randomize colors and inter-stimulus times, making it difficult for participants to anticipate the timing of the following visual stimulus and the direction of the targeted motor response.

Accuracy was calculated using the following formula: Accuracy (%) = ((Total number of stimuli−(missed stimuli + multiple attempts needed + decision errors + balance errors))/Total number of stimuli) x 100. The definitions for each error are provided in Table 2. Each experimental trial was filmed with a video camera (Handycam 1,080 50i, HDR-CX105E, Sony Corporation, Japan) to manually analyze accuracy based on videorecording. When reviewing the RBT videos, raters recorded instances where participants missed stimuli, required multiple attempts, made decision errors, and exhibited balance errors.

**Table 2:** Errors definitions – Verscheuren et al. (79).

Error	Definition
Missed stimulus	The participant failed to extinguish the LED light
Multiple attempts	The participant reached from the standardized position but failed to extinguish the LED light the first time. The participant needed two or more attempts
Decision error	The participant initiated a movement in the wrong direction
Balance error	- The participant did not start from the standardized position at the stimulus onset - The participant is trying to find balance during the reach - The participant needs to put a hand or foot on the floor - The participant steps off the YBT Test kit - The participant is not able to keep the hands on the hips - The participant lifts the forefoot or heel off the testing surface

YBT, Y-Balance test.

**Figure 3:**
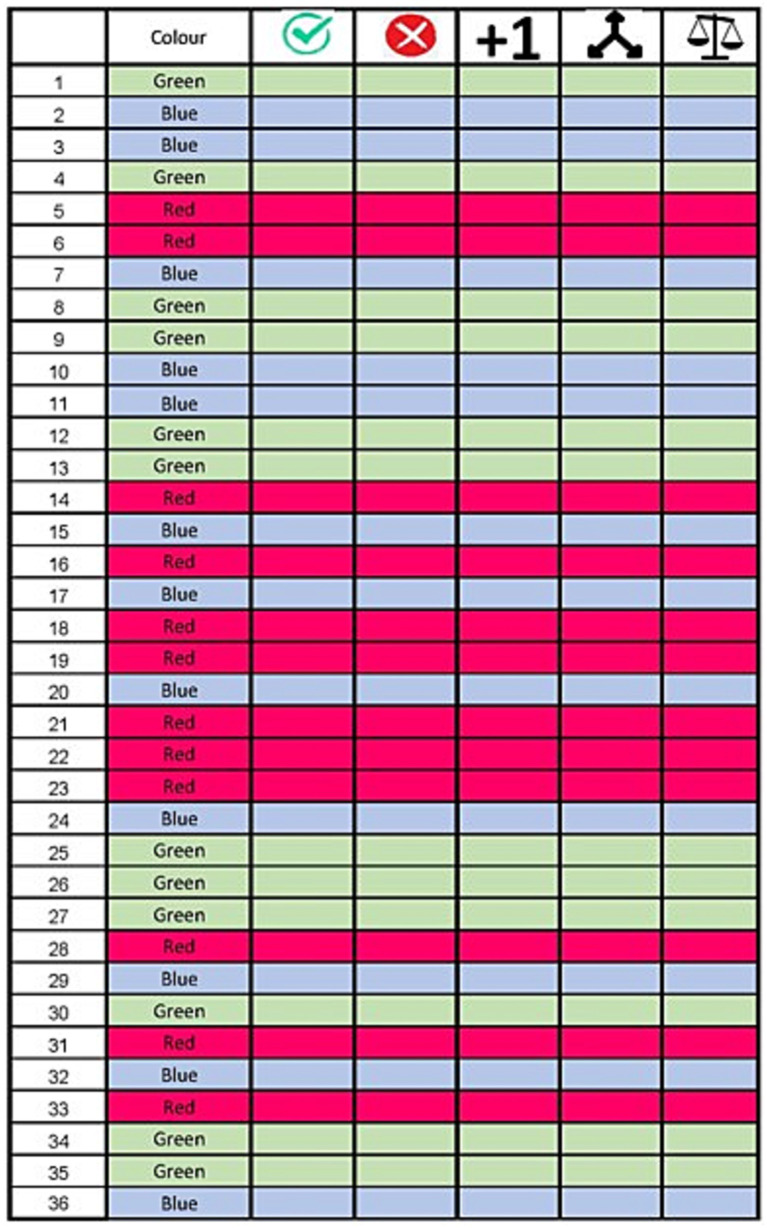
Reactive balance test score sheet. The first column indicates the stimulus number. The target color is indicated by the color on the score sheet and stated in column 2. The following columns indicate the possible results on a stimulus: successful, missed, multiple attempts, decision error and balance error (in order).

To score the accuracy of the participant in real-time, we have created a score sheet with columns representing stimulus number, target light, and four error types. The score sheets were adapted to the sequence of the stimuli used in each trial. Missed lights were given priority over stimuli that required multiple attempts or contained balance and decision errors. The VMRT was corrected for accuracy by removing the corresponding VMRT values from the original Fitlight^™^ dataset. The mean VMRT was obtained by only counting the correct extinguished LED lights. The score sheet can be found in Figure 3.

### 2.3 Raters

One rater with a Master of Science degree in physiotherapy and rehabilitation sciences, and the other raters were master’s students (E.D. and A.W.) in physiotherapy and rehabilitation sciences, with experience in YBT and RBT procedures evaluated RBT accuracy and VMRT. Raters were blinded to each other’s evaluations. Test-retest reliability was determined by comparing rater 1 ratings from both experimental trials, intra-rater reliability was computed by comparing ratings of rater 1 from the second experimental trial. Inter-rater reliability was determined by comparing rater 1 and rater 2 ratings from the second experimental trial, and score sheet method reliability was compared using rater 2 ratings from both experimental trials and comparing them to rater 3 ratings.

### 2.4 Statistical analysis

Statistical analyzes were performed using the RStudio software version 4.2.2 (2022-10-31 ucrt). The mean, standard deviation and 95% confidence interval were calculated for accuracy and VMRT to illustrate the participants’ characteristics. Reliability calculations were applied to the data of the self-reported CAI side confirmed by the lowest CAIT score. The Shapiro Wilk test was used to assess the normality of the data distribution, and this was supplemented by visual inspection using histograms and QQplots (87). To describe all reliability measures, we used the following indexes: Pearson r, intraclass coefficients (ICC), standard error of measurement (SEM), standard error of prediction (SEP) and minimal detectable change (MDC) (88–98). Additionally, we reported Bland–Altman plots to graphically render the agreement between two raters or ratings and the limits of the agreement (LOA) (99, 100). A paired t-test was used to detect any systematic biases between raters, ratings, or trials.

The guidelines of Koo and Li (88) were employed to select the appropriate model, type, and definition of ICC. ICC values less than 0.5 indicate poor reliability, values between 0.50 and 0.75 indicate moderate reliability, values between 0.75 and 0.90 indicate good reliability and values greater than 0.90 indicate excellent reliability (101). We interpreted Pearson r correlations between 0.1 and 0.3 as weak, between 0.3 and 0.5 as moderate and above 0.5 as strong. The statistical significance level was set at *p* < 0.05 for all statistical analyzes.

A two-way random absolute agreement, single-rater model (ICC 2.1), was used to estimate the inter-rater reliability. The test-retest and intra-rater reliability were calculated using a two-way mixed effect, consistency, single-rater model (ICC 3.1) as ICC equivalent.

## 3 Results

An overview of the relationship between variables (Pearson r and ICC), variability measures (SEM and SEP) and the ability of the RBT to detect true changes in score (MDC) for all reliability outcome measures are shown in Table 3.

**Table 3:** Reliability outcome measures overview.

	Pearson *r*	ICC [95% CI]		SEM	SEP	MDC	MEAN DIFF	LOA
Video-based analysis								
Test-retest reliability								
ACC VMRT	0.612 0.633	0.616 0.600	[0.305;0.808] [0.284;0.799]	6.6% 59 ms	8.4% 74 ms	18.2% 162 ms	−1.4% 19 ms	[−23;20] [−149;187]
Intra-rater reliability								
ACC VMRT	0.816 0.802	0.815 0.800	[0.635–0.911] [0.608–0.904]	4.4% 45 ms	5.9% 60 ms	12.1% 123 ms	−2.7% 4 ms	[−15;11]* [−167;175]
Inter-rater reliability								
ACC VMRT	0.738 0.875	0.690 0.868	[0.419–0.846] [0.731–0.937]	4.6% 40 ms	5.9% 54 ms	12.6% 110 ms	1.2% 9 ms	[−12;14] [−157;176]
Score sheet method								
Test-retest reliability								
ACC VMRT	0.696 0.598	0.695 0.585	[0.413–0.856] [0.245–0.796]	4.7% 69 ms	6.1% 87 ms	13.0% 191 ms	0.1% 38 ms	[−18;18] [−163;238]
Inter-rater reliability								
ACC VMRT	0.831 0.959	0.822 0.958	[0.651–0.915] [0.911–0.981]	3.1% 23 ms	4.2% 32 ms	8.5% 64 ms	1.4% 7 ms	[−14;17] [−161;175]

^*^Statistically significant; RBT, Reactive balance test; VMRT, Visuomotor response time (in ms); ACC, accuracy (in %); ICC [95%CI], 95% Confidence Interval of the Intraclass Correlation Coefficient; LOA, Limit Of Agreement; MEAN DIFF, Mean Difference between raters/ratings; SEM, Standard Error of Measurement; SEP, Standard Error of Prediction; MDC, Minimal Detectable Change.

### 3.1 Video-based RBT assessment

Patients with CAI scored an average accuracy of 81.20% (± 9.73%; CI: 77.27–85.13%) and 80.45% (± 10.60%; CI: 76.26–84.65%) for the first and second experimental trials, respectively. Corresponding VMRT were 779 ms (± 133 ms; CI: 725–832 ms) and 770 ms (± 93 ms; CI: 733–806 ms). The accuracy data were normally distributed. VMRT was normally distributed for the first, but not for the second experimental trial. The first and second trials showed a correlation of 0.612 for accuracy and 0.633 for VMRT. Similar ICC measures were found at 0.609 (0.297–0.804) for accuracy and 0.594 (0.276–0.795) for VMRT between both trials. We computed the SEM and SEP based on the 95% confidence interval (CI) and standard deviation (SD) for both outcomes: 6.6 and 8.4% for accuracy and 59 ms and 74 ms for VMRT. The MDC was 18.4% for accuracy and 164 ms for VMRT.

Intra-rater reliability was determined with a two-week interval by rater 1. Mean accuracy in the second evaluation was 79.42% (± 10.17%, CI: 75.40–83.45%) and mean VMRT was 761 ms (± 100 ms; CI: 721–800 ms). Both ratings were correlated with a Pearson r of 0.816 and ICC of 0.815 (0.635–0.911) for accuracy. Similar measures were found between the two VMRT ratings. The Pearson r was 0.802, and the ICC was 0.800 (0.608–0.904). The following variability measures were computed from both ratings for both outcomes. SEM was 4.37% and SEP 5.89% for accuracy, while it was 45 ms and 60 ms, respectively, for VMRT. The MDC of both outcomes were 12.11% and 123 ms.

Rater 2 estimated a mean accuracy of 83.33% (± 8.19%; CI: 80.09–86.57%) and a mean VMRT of 766 ms (± 109 ms; CI: 723–809 ms). When comparing both raters, we calculated the following correlation: 0.738 and 0.875 for accuracy and VMRT, respectively. While the degree of association for the accuracy outcome was reflected in an ICC of 0.690 (0.419–0.846), the VMRT ICC was 0.868 (0.731–0.937). The variability measures between both raters for accuracy were 4.56 and 5.93% for the SEM and SEP, while the same outcome measures were 40 ms and 54 ms, respectively, for the VMRT calculations. The MDC was 12.65% and 110 ms between both raters.

### 3.2 Score-sheet-based RBT assessment

To determine test-retest reliability, rater 2 rated the first and second experimental trials using the score-sheet assessment method. Patients with CAI scored an average accuracy of 83.4% (± 8.99%; CI: 79.7–87.3%) and 84.8% (± 8.5%; CI: 81.4–88.1%) for the first and second experimental trial, respectively. The accuracy outcome measure was normally distributed. To describe the test-retest reliability of the accuracy measure we have calculated the pearson *r* (= 0.696) and ICC (= 0.695 [0.413–0.856]). The SEM and SEP of the second experimental trial were 4.71 and 6.13%, respectively. The MDC was 13.04%. VMRT was also normally distributed. The average VMRT across participants was 779 ms (± 135 ms; CI: 722-836 ms) and 785 ms (± 107 ms; 743–828) for both experimental trials.

To determine the inter-rater reliability of the RBT using the score-sheet method, rater 3 outcomes were compared with rater 2 outcomes. Patients with CAI scored an average accuracy score of 85.6% (± 7.3%; CI: 82.7–88.5%) and VMRT as 781 ms (± 112; CI: 737–826). Accuracy data was normally distributed whereas VMRT data was not for both raters. Rater 3 ratings correlated with rater 2 with a pearson r of 0.831 for accuracy and 0.959 for VMRT. ICC were 0.823 (0.652–0.915) and 0.959 (0.913–0.981) for both outcomes. The raters showed a SEM of 3.1%, SEP of 4.2% and MDC of 8.5% for accuracy. The same reliability measures for VMRT were 23 ms, 32 ms and 63 ms, respectively.

## 4 Discussion

This study is the first to determine the test-retest, intra- and inter-rater reliability of a neurocognitive functional performance test in patients with CAI. The reliability of the video-based analysis is considered moderate to good. The data indicates the VMRT performance was more robust than the accuracy measure across the trials. When the LOA are compared with the MDC, the data indicates the RBT is more precise and sensitive to changes than the raters’ score. We are also the first to report on a RBT score sheet’s test-retest and inter-rater reliability. The newly designed score sheet was created to make it more time-efficient to evaluate the accuracy of the RBT and, thus, facilitate the use of the RBT in clinical practice. The moderate to excellent reliability measures of the score sheet method show that this novel scoring system can be used as a valid substitute for video analyzes.

The video analysis showed that the test-retest reliability for accuracy and VMRT was moderate. However, there may still be some variability in scores that cannot be attributed to chance or measurement error, which may stem from individual differences, rater bias, or administration issues. It is worth noting, however, that its MDC is relatively large and, thus, that the RBT is not sensitive to detect small changes in performance over time.

Furthermore, when multiple raters are involved, the RBT’s accuracy ratings may be influenced by chance or by variations in the raters’ rigor. The observed variability is more likely due to chance as the SEM is relatively small (4.6%). The high MDCs (12.7%) and significant LOA (−15%;11%) might explain its moderate reliability for accuracy, however. VMRT outcomes were more robust when rated by multiple raters.

The RBT maintains consistency when assessing performance over time. Its wider LOA range, however, raises the possibility that accuracy ratings may change significantly over time. In contrast, the score sheets show good to excellent reliability for inter-rater and test-retest comparisons. However, some unexplained variability over time remains present.

Under the current conceptualisations of the PROMIS health organization to be able to use a measuring instrument, acceptable minimal ICC standards for reliability coefficients are ≥0.70 for group comparisons and between 0.90 and 0.95 for individual comparisons (102). When we apply these recommendations to the current study, the test-retest reliability results of the RBT using either scoring method do not meet the criteria for group analyzes or follow-up measures in scientific research. These results are in contrast with the study of Tassignon et al. (80), which tested the reliability of the RBT in healthy recreational athletes and found an ICC ≥ 0.70. However, several factors may contribute to the increased variability of the results, and these will be explored in the limitation section. Additionally, other conclusions in line with the study of Tassignon et al. can be drawn from this study: the reliability increases if the same rater scores the RBT and another rater can vouch if it is not practically possible to always use the same rater, especially when interested in the VMRT outcome.

Our results imply there may be room for improvement when designing a reliable neurocognitive functional performance test. Changes in the test administration process may reduce variability and increase sensitivity to detect small changes in performance over time. The findings also highlight the importance of using different reliability outcome measures, such as the LOA, to interpret results accurately and account for variability between test sessions or raters. Overall, this emphasizes the need for continued research and refinement of assessment methods to measure neurocognitive components accurately and reliably in functional performance tests.

Despite the need for caution when interpreting results, the moderate to good reliability of the RBT, and, particularly, the good to excellent reliability of the score sheet method suggest the test can be useful to assess certain aspects of cognitive function in a clinical setting. However, it is important to keep in mind the limitations of the test, especially when the goal is to detect small changes over time. Thus, while the RBT and the newly developed score sheet method may be valuable tools for clinicians, continued efforts are necessary to improve their accuracy and sensitivity in measuring cognitive function and dynamic balance ability.

### 4.1 Limitations and future research

It is important to keep in mind that reliability is a measure of consistency, not accuracy. Even if there is some variability in the scores due to rater differences, this does not necessarily mean that the measure itself is inaccurate or invalid. It means that there may be some inconsistency in how the measure is being administered or scored across different raters or ratings which could potentially impact the interpretation of the scores. This is especially relevant as even small differences between accuracy ratings can have a significant impact on the overall test score because the MDC is relatively high (8.5–18%). Therefore, further defining errors could minimize rater variability and improve the reliability of the RBT test. Another potential reason to explain this increased variability compared to healthy recreational athletes (80), might be the inherent increased variability in postural control strategies used by patients with CAI (103–108). It might be driven by the neuroplastic changes they experience related to their ligamentous ankle injuries (109, 110). Additionally, the included participants had quite a large standard deviation in IPAQ score (SD = 2322MET), which is more than 5 times the standard deviation of the IPAQ score of the participants of the study of Tassignon et al. This is relevant as the ICC estimates depend on the characteristics of the population and may explain the increased variability.

Despite these factors, the RBT still shows strong reliability measures regardless of reliability type. The SEM for RBT outcomes is small, especially considering the degree of variability of the data (VMRT: 40–59 ms, accuracy: 4.4–6.6%). This suggests an increased precision and validity of the RBT. In addition, the mean difference between tests, ratings or raters is even smaller than the SEM (VMRT: 4–19 ms, accuracy: −2.7–1.2%) indicating very little bias and an increased confidence of the results between measurements. The RBT may not be best suited for group analyzes in research according to the PROMIS health organization, but it remains a useful tool for patients with CAI. The addition of neurocognitive load to functional performance tests brings these exercises closer to the sports context due the demand of being able to respond and adapt to a changing environment (60, 79, 111). Evidence emerged suggesting that lower neurocognitive performance is associated with an increased risk of ankle injury (112, 113). As demonstrated in a systematic review, patients with CAI (110) show adaptations related to their injury and could benefit from neurocognitive training to cope with neuroplasticity related to ligamentous ankle injuries. However, there are no studies reporting on the benefits of neurocognitive training on LAS injury risk yet. Future studies should investigate whether neurocognitive training could positively influence and, thus, reduce injury risk as further research is needed to understand the different modalities of this approach.

## 5 Conclusion

The RBT is a neurocognitive functional performance test which can be analyzed either video-based or via score sheet method. The video-based analyzes revealed moderate test-retest reliability, but good intra-rater reliability in patients with CAI. When multiple raters evaluate the RBT performance, the VMRT outcome is more reliable than the accuracy outcome. Additionally, the RBT shows robust SEM and mean difference measures. The score sheet method showed similar reliability: over time the reliability was moderate, however, the reliability between raters was good to excellent. Also, the RBT can be a useful tool to assess and monitor balance in patients with CAI as it incorporates neurocognitive elements: environmental perception and decision-making.

## Data availability statement

The raw data supporting the conclusions of this article will be made available by the authors, without undue reservation.

## Ethics statement

The studies involving humans were approved by Ethics Committee of the UZ Brussel/Vrije Universiteit Brussel. The studies were conducted in accordance with the local legislation and institutional requirements. The participants provided their written informed consent to participate in this study.

## Author contributions

AM: Writing – original draft, Writing – review & editing. EL: Writing – original draft, Writing – review & editing. JV: Writing – original draft, Writing – review & editing. KP: Writing – original draft, Writing – review & editing. RM: Writing – original draft, Writing – review & editing. BR: Writing – original draft, Writing – review & editing. BT: Writing – original draft, Writing – review & editing.
